# Frequency of ABO Blood Group in Pregnant Women and Its Correlation With Pregnancy-Related Complications

**DOI:** 10.7759/cureus.14487

**Published:** 2021-04-14

**Authors:** Reshma Sajan, Sajan Lal, Sarah Kazi, Anum Sultan, Saima Ismail, Gulraiz Khanzada

**Affiliations:** 1 Obstetrics and Gynecology, Civil Hospital Karachi, Karachi, PAK; 2 Radiology, Dr. Ziauddin Hospital, Karachi, PAK

**Keywords:** abo blood group, pregnancy-related complications, preeclampsia, gdm, preterm delivery, lbw, sga

## Abstract

Introduction

The ABO blood group type has been considered an independent risk factor in the occurrence of pregnancy-related complications leading to significant morbidity and mortality in pregnant mothers and neonates. This study aims to determine the maternal ABO blood group and its correlation with pregnancy-related complications.

Methods

We analysed data of 497 pregnant female patients aged between 25 and 40 years who presented with a gestational age of > 14 weeks from June 1, 2020, to November 30, 2020. Patients’ age, parity, gestational age at the first visit, body mass index (BMI) at the first visit, ABO blood group, gestational age at delivery, mode of delivery, birth weight of neonate, and pregnancy-related complications including preeclampsia, gestational diabetes mellitus (GDM), preterm delivery, low birth weight (LBW), and small for gestational age (SGA) infants were evaluated.

Results

The mean age of 497 patients was 27.6 (27.62 ± 3.35) years. Mean BMI was 22.7 (22.7 ± 3.1 kg/m^2^), parity was 1.85 (1.85 ± 2.3), gestational age at first visit was 23.19 (23.19 ± 3.4) weeks and gestational age at delivery was 37.0 (37.0 ± 2.6) weeks. There were 205 (41.25%) spontaneous vaginal delivery and 292 (58.75%) cesarean sections. The average birth weight of the neonate was 2684.31 ± 622.4 g. Preeclampsia was observed in 107 (21.53%), GDM in 17 (3.42%), and preterm delivery in 154 (30.99%) women. Considering the neonatal outcome, 124 (24.95%) babies had LBW and 49 (9.86%) were SGA. The rate of preeclampsia and GDM was not statistically significant among different blood groups while the rate of preterm delivery, LBW, and SGA was significant among women with different blood groups.

Conclusion

We conclude that the ABO blood group is associated with maternal and neonatal pregnancy-related complications when considering the risk of preterm delivery, LBW, and SGA but not with GDM and preeclampsia. This finding will help clinicians to identify the patients at risk of developing pregnancy-related complications and hence, to take timely and appropriate measures.

## Introduction

Initially discovered in 1900 by Karl Landsteiner, the ABO blood group system categorizes the human blood groups into A, B, O, and AB based on the presence of naturally occurring antigens on red blood cells and serum antibodies [1]. Having a primary role in blood transfusion and organ transplantation, recent advances have also elucidated the importance of ABO blood groups in the pathogenesis of various disorders such as diabetes mellitus, cardiovascular, neoplastic, and infective disorders as well as in gastric and duodenal ulcers. The role of Rh compatibility screening in erythroblastosis fetalis has already been established [2].

Pregnancy-related complications are a major health concern worldwide leading to significant morbidity and mortality in pregnant mothers and neonates [3]. It has been reported that approximately 1%-5% of patients develop serious pregnancy-related adverse complications [4]. These include preeclampsia, gestational diabetes mellitus (GDM), HELLP (hemolysis, elevated liver enzymes, and low platelets) syndrome, preterm delivery, small for gestational age (SGA), and low birth weight (LBW) babies [3,4]. The ABO blood group type has been considered an independent risk factor in initial studies resulting in the occurrence of pregnancy-related complications. Various studies have been conducted to identify the association of ABO blood groups with pregnancy-related complications in pregnant patients and neonates; however, there is a wide variation and inconsistency in their results [3]. According to a study by Zhang et al., the prevalence of GDM ranges from less than 1% to 28%, thus showing significant variance [5]. Similarly, the wide discrepancy is also seen in the results of studies evaluating the association of preeclampsia with ABO blood group type. Some authors reported an increased incidence of preeclampsia in AB blood groups as compared to type O blood group while others showed no association between blood group type and preeclampsia [6].

Taking into consideration the conﬂicting results from prior studies and no data available on this subject in Pakistan, there is a dire need for research on this subject in our population. Our study aims to determine the maternal ABO blood group and its correlation with pregnancy-related complications. In the case of significant adverse outcomes in particular blood groups, we can devise a strategy of close outpatient follow-up in patients with such blood groups so that factors contributing to an adverse pregnancy outcome can be detected early and timely strategies to correct it can be made to improve the pregnancy outcome.

## Materials and methods

This was a retrospective study that included all pregnant female patients aged between 25 and 40 years who presented with a gestational age of >14 weeks as confirmed by ultrasound examination, to the Department of Obstetrics and Gynecology, Civil Hospital, Dow University of Health Sciences, Karachi, from June 1, 2020, to November 30, 2020, and was conducted after obtaining the approval of the institutional ethics committee (IEC). Patients who did not give informed consent and had known co-morbidities including chronic hypertension assessed clinically with history and raised blood pressure (BP) more than 140/90, overt diabetes assessed by raised HbA1c levels of more than 8, renal failure assessed by raised creatinine levels, decreased glomerular filtration rate (GFR) of less than 30, known hyperthyroidism confirmed by history, congenital fetal anomalies assessed by antenatal ultrasound and Rh-negative blood type were excluded. The evaluation of ABO blood groups of patients was done by performing blood serology analysis laboratory test for the presence or absence of red blood cell A and B antigens and serum anti-A and anti-B antibodies. The laboratory results for each test were certified by a consultant pathologist with an experience of more than five years. Pregnancy-related complications were divided into maternal and neonatal. Maternal adverse pregnancy outcomes were categorized as follows:

- Pre-eclampsia: It was defined as having hypertension (BP > 140/90 mmHg) along with proteinuria (urinary proteins ≥300 mg/24 hr) after 20 weeks of gestational age.

- Gestational diabetes mellitus: It was defined as raised blood glucose levels taken after one and three hours of glucose administration on the glucose challenge test. Blood glucose levels of more than 140 mg/dL at one hour and two or more high values of blood glucose at three hours were labeled as raised glucose levels on the oral glucose tolerance test.

- Preterm delivery: It was defined as the delivery of a fetus with gestational age less than 37 weeks as assessed by ultrasound.

Neonatal complications included low birth weight, with neonatal birth weights <2500 g, and small for gestational age, with neonatal birth under the 10th percentile for that particular gestational age.

Data collection was performed by filling a predesigned performa that included maternal age, parity, gestational age at the first visit, body mass index (BMI) at the first visit, ABO blood group, gestational age at delivery, mode of delivery, birth weight of newborn and adverse pregnancy outcomes. Patient data were analysed using Statistical Package for the Social Sciences (SPSS), Version 21 (IBM Corp., Armonk, NY). Frequencies and percentages were computed for qualitative variables such as mode of delivery, ABO blood group (A, B, AB, O), and adverse pregnancy outcomes, i.e. preeclampsia, GDM, preterm delivery, LBW, and SGA infants. Quantitative variables like maternal age, BMI, parity, and gestational age at delivery were presented as means ± SDs. Effect modifiers like age, parity, BMI, gestational age at delivery, mode of delivery and blood groups were controlled through stratification. A post-stratification chi-square test was applied and P-value ≤ 0.05 was considered significant.

## Results

**Figure 1:**
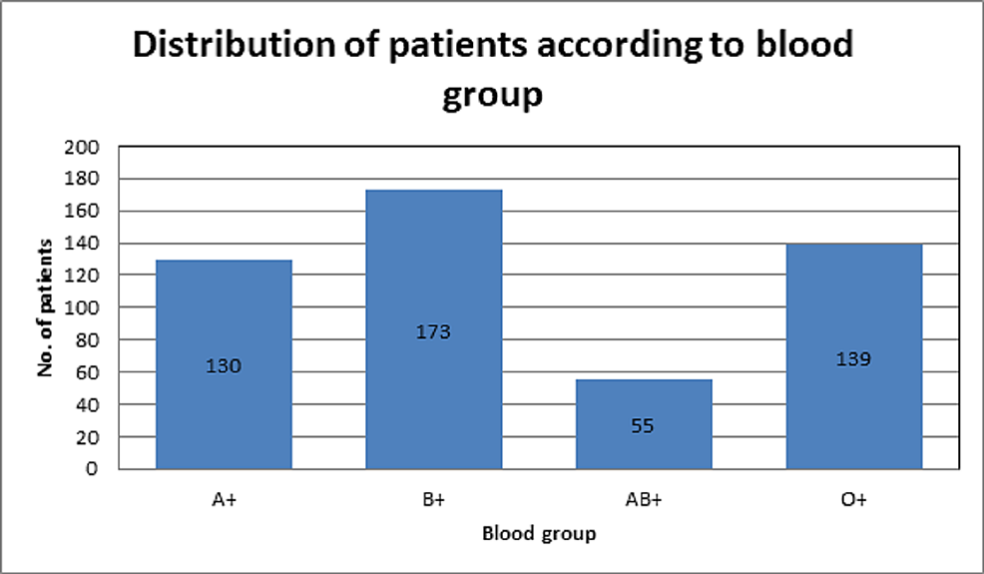
Distribution of patients according to the ABO blood group

A total of 497 pregnant female patients with a gestational age of more than 14 weeks were included in this study. The mean age of the patients was 27.6 (27.62 ± 3.35) years. The mean BMI was 22.7 (22.7 ± 3.1 kg/m^2^), parity was 1.85 (1.85 ± 2.3), gestational age at the first visit was 23.19 (23.19 ± 3.4) weeks and gestational age at delivery was 37.0 (37.0 ± 2.6) weeks. There were 205 (41.25%) spontaneous vaginal deliveries and 292 (58.75%) cesarean sections. The average birth weight of the neonate was 2684.31 ± 622.4 g. The ABO blood group status of women is shown in Figure 1.

**Figure 2:**
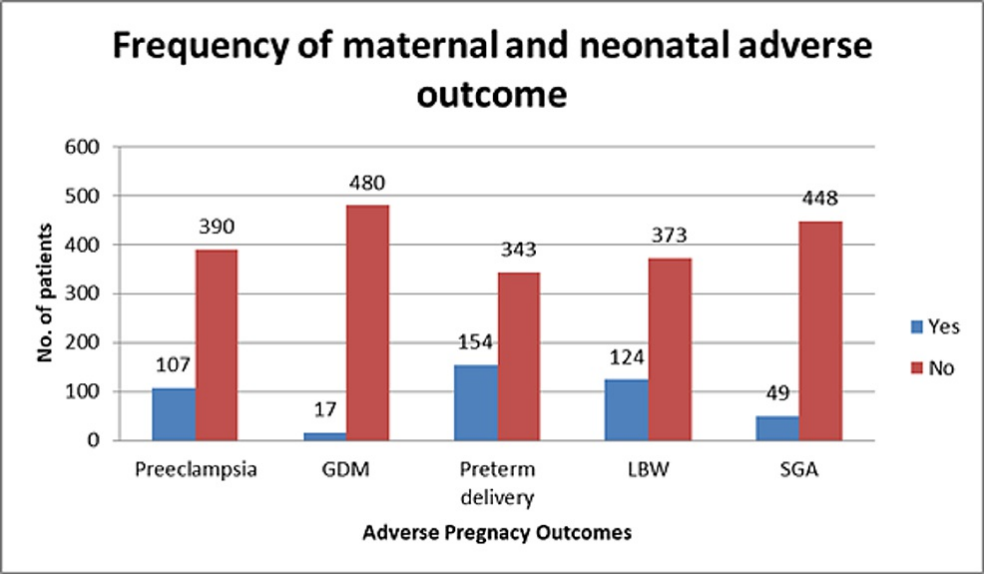
Frequency of maternal and neonatal pregnancy-related complications GDM, gestational diabetes mellitus; LBW, low birth weight; SGA, small for gestational age

The frequency of different maternal and neonatal pregnancy-related complications including preeclampsia, GDM, preterm delivery, LBW, and SGA is depicted in Figure 2.

Regarding maternal outcomes, preeclampsia was observed in 107 (21.53%), GDM in 17 (3.42%), and preterm delivery in 154 (30.99%) women; while considering the neonatal outcomes, 124 (24.95%) babies had LBW and 49 (9.86%) were SGA. The rate of preeclampsia and GDM was not statistically significant among different blood groups while the rate of preterm delivery, LBW, and SGA was significant among women with different blood groups as shown in Table 1.

**Table 1:** Comparison of pregnancy-related complications between different maternal blood groups GDM, gestational diabetes mellitus; LBW, low birth weight; SGA, small for gestational age

Adverse Pregnancy Outcome	Blood Groups				Chi-Square	P-Value
	A+ (n=130)	B+ (n=173)	AB+ (n=55)	O+ (n=139)		
Preeclampsia	22 (16.9%)	37 (21.4%)	12 (21.8%)	36 (25.9%)	3.209	0.631
GDM	0 (0%)	9 (5.2%)	3 (5.5%)	5 (3.6%)	6.969	0.073
Preterm delivery	55 (42.3%)	46 (26.6%)	10 (18.2%)	43 (30.9%)	13.573	0.004
LBW	43 (33.1%)	43 (24.9%)	5 (9.1%)	33 (23.7%)	12.08	0.007
SGA	9 (6.9%)	29 (16.8%)	3 (5.5%)	8 (5.8%)	14.37	0.002

The rate of preeclampsia and SGA was significantly high in women aged 25-30 years with a significant P-value of 0.04 and 0.02, respectively. P-value was not statically significant for GDM, preterm delivery, and LBW when comparing women aged 25-30 and 31-40 years. The rate of GDM was high in those women who had a BMI >25 kg/m^2^ (P-value 0.0005) while the rate of LBW and SGA was significantly high in those with a BMI ≤25 kg/m^2^. Adverse pregnancy outcomes were also compared concerning parity, gestational age at the time of delivery and mode of delivery. A significant P-value (0.001) was seen for SGA in low-parity patients (parity 0-1). Preterm delivery, LBW and SGA infants were more frequently encountered in patients with gestational age ≤36 weeks at the time of delivery with significant P-values. C-section was the predominant mode of delivery seen in 292 patients with adverse pregnancy outcomes. Most of the patients with GDM had undergone C-section, with a significant P-value of 0.01.

## Discussion

The role of blood group type in the ABO grouping system as a contributing factor in the pathogenesis of multiple diseases has been established. Several studies have been done to elucidate the impact of ABO blood group on pregnancy and its related complications in a mother and newborn infant. There is a wide variation in the frequency of different phenotypes including A, B, AB, and O among different populations based on their ethnicity across the globe [7]. In Pakistan, many researches have been done in different regions of the country showing variations in blood groups among different ethnic populations. From these studies, it is evident that the relative frequency of ABO and Rhesus blood groups does not deviate from the typical pattern that is group B being most common followed by groups O, A, and AB in order of prevalence [8]. A total of 497 pregnant patients aged 25 to 40 years were included in this study and the frequency of maternal ABO blood group and its relationship with pregnancy-related complications were determined. Regarding ABO blood group status, the results of our study showed that 26.16% of women were A+, 34.8% were B+, 11.07% were AB+ and 27.9% were O+. These results were similar to the results of previous studies [8].

Regarding the correlation between blood group type and its role in the development of preeclampsia, there is a wide variance in the results of previous studies. In the studies by Lee et al. and Franchini M et al., patients with blood group AB are most prone to develop preeclampsia and severe eclampsia [6,9]. They stated that the non-O blood group has a modest risk to develop preeclampsia as compared to the O group. Thus, O group holds a protective effect for preeclampsia. Similar results are shown in the study by Avci et al. [10]. Beyazıt et al. stated in their study that there was no association between different blood groups and preeclampsia. In our study, regarding the maternal outcome, preeclampsia was observed in 21.53% of women with no statistical significance between different blood groups. The results of our study are similar to the results of the study by Beyazıt et al. [3].

Few studies have been done in the past to analyse the association of blood group with the development of GDM in pregnant women. It has been stated that multiple factors such as genetic variation, intestinal flora and inflammatory markers may contribute to the development of GDM. Zhang et al. in their study showed that blood groups A and B were associated with an increased risk of GDM; however; blood group AB has been a protective factor in its development [5]. In another study, Donma found that most patients with GDM had blood group O and hence found it to be the risk factor [11]. In our study, regarding the maternal outcome, GDM was seen in 17 patients (3.42%) and no statistical difference was seen between different blood groups. The results of our study are in discordance with the results of previous studies.

The results of our study show that 30.99% of patients had preterm delivery; while considering the neonatal outcome, 24.95% of babies had LBW and SGA was 9.86%. In our study, the rate of preterm delivery, LBW, and SGA was statistically significant among women with different blood groups. Preterm delivery was found in 42.3% in A+, 30.9% in B+, and 26% in the B+ blood group. LBW was found to be 33.1% in A+, 24.9% in B+, and 23.7% in the O+ blood group. Phaloprakarn and Tangjitgamol in their study found that A blood type was a significant risk factor for coincidental events of SGA [12]. A prospective case-control study conducted by Clark and Greer showed no association between intrauterine growth retardation and ABO genotype or phenotype [13], while in our study, SGA was found to be significant in the B+ group at 16.8%.

The limitations of our study are that Rh-negative blood group phenotype patients were not included; consequently, the impact of this particular blood type was not determined. Moreover, it was a single-center study with a limited sample size.

## Conclusions

We conclude that the ABO blood group is associated with maternal and neonatal adverse pregnancy outcomes when considering the risk of preterm delivery, LBW, and SGA, but not with GDM and preeclampsia. This finding will help clinicians to identify the patients at risk of developing adverse pregnancy outcomes and hence, to adopt appropriate timely strategies to improve the outcome.
